# Emotional Flexibility, Biomarkers, and Cognitive Resilience in Latino Adults at Midlife

**DOI:** 10.1093/geroni/igaa057.2077

**Published:** 2020-12-16

**Authors:** Erica Diminich

**Affiliations:** Stony Brook University, Stony Brook, New York, United States

## Abstract

A growing body of evidence underscores the important role of emotional responding and emotional flexibility in healthy adaptation. Considerable research further demonstrates that being flexible in how one copes and regulates emotions when faced with stressful events is paramount for healthy aging. However, the adaptive benefits of emotional flexibility have not been studied in Latina/o’s, despite converging evidence indicating that Latina/o’s report greater symptoms of depression and anxiety in the context of exposure to potentially traumatic events and stress. We demonstrate across two studies, how the ability to regulate emotional responses is associated with cognitive health in a community-based population of Latinos and a cohort of Latino responders from the World Trade Center attacks on 9/11. Furthermore, given that individual differences in emotional flexibility predict cognitive decline, we present data examining the utility of plasma biomarkers of pre-clinical Alzheimer’s disease and neuropathy as diagnostic screeners of cognitive functioning and health.

